# Clinical and Radiological Characteristics of Lumbosacral Lateral Disc Herniation in Comparison With Those of Medial Disc Herniation

**DOI:** 10.1097/MD.0000000000002733

**Published:** 2016-02-18

**Authors:** Jung Hwan Lee, Sang-Ho Lee

**Affiliations:** From the Department of Physical Medicine and Rehabilitation (JHL) and Department of Neurosurgery, Wooridul Spine Hospital, Seoul, Korea (SHL).

## Abstract

Lateral disc herniation (foraminal and extra foraminal) has clinical characteristics that are different from those of medial disc herniation (central and subarticular), including older age, more frequent radicular pain, and neurologic deficits. This is supposedly because lateral disc herniation mechanically irritates or compresses the exiting nerve root or dorsal root ganglion inside of a narrow canal more directly than medial disc herniation. The purpose of this study was to investigate clinical and radiological characteristics of lateral disc herniation in comparison with medial disc herniation. The 352 subjects diagnosed with localized lumbosacral disc herniation and followed up for at least 12 months after completion of treatment were included and divided into medial and lateral disc herniation groups, according to the anatomical location of the herniated disc in axial plain of magnetic resonance image. Clinical and radiological data were obtained and compared between the two groups. The lateral group included 74 (21%) patients and the medial group included 278 (79%). Mean age of the lateral group was significantly higher than that in the medial group. The lateral group showed a significantly larger proportion of patients with radiating leg pain and multiple levels of disc herniations than the medial group. No significant differences were found in terms of gender, duration of pain, pretreatment numeric rating scale, severity of disc herniation (protrusion and extrusion), and presence of weakness in leg muscles. The proportion of patients who underwent surgery was not significantly different between the 2 groups. However, the proportion of patients who accomplished successful pain reduction after treatment was significantly smaller in the lateral than in the medial group. In conclusion, patients with lateral disc herniation were older and had larger proportion of radiating leg pain than those with medial disc herniation. Lateral disc herniation was more associated with multiple disc herniations and worse clinical outcomes after treatment than medial disc herniation.

## INTRODUCTION

Incidence of lateral disc herniation is lower than that of medial disc herniation and has been reported to account for 7% to 12% of all lumbosacral disc herniations.^1–3^ Lateral disc herniation has different clinical characteristics from medial disc herniation. Patients with lateral disc herniation can manifest with more severe clinical symptoms, including severe radicular pain, or more frequent motor and sensory neurological deficits than those with medial disc herniation,^4^ because the herniated disc fragment is located in or around a narrow root foramen, through which nerve root passes, resulting in direct compression of dorsal root ganglion which is a pain sensitive structure.^2,5^ Additionally, clinical outcomes of lateral disc herniation after transforaminal injection or surgery were found to be worse than those of medial disc herniation.^5,6^ To our knowledge, no study has identified the clinical and radiological characteristics of patients with lateral disc herniation in comparison with medial disc herniation. The purpose of this study was to investigate clinical and radiological characteristics of lateral (foraminal and extraforaminal) disc herniation in comparison with medial (central and subarticular) disc herniation, which might provide useful information about lateral disc herniation, helping in diagnosis and prediction of prognosis after treatment in these patients.

## METHODS

This study was approved by Institutional Review Board of our hospital. This was retrospective study so that informed consent was not required. Patients who visited our hospital with chief complaint of back and/or radiating leg pain and were diagnosed with lumbosacral disc herniation through clinical and radiological evaluation, including lumbosacral magnetic resonance image (MRI) from January 2014 to June 2014, were chosen. Disc herniation was defined as a localized displacement of disc material beyond the limits of the intervertebral disc space. “Localized” was defined as less than 25% of the disc circumference.^7^ Patients with bulging disc involving greater than 25% of its circumference, spinal stenosis, severe disc degeneration, significant instability, spinal tumor, prominent scoliosis (Cobb angle >15°), and previous lumbar surgery were excluded. Patients diagnosed with vertebral bone fractures and infectious diseases, such as discitis or spondylitis, evidenced by MRI were also excluded. Among the 635 patients diagnosed with disc herniation, those with sequestered and migrated disc herniations were excluded because anatomical classification of these types in axial plane is difficult to be determined. Disc herniation was divided into central, subarticular, foraminal, and extra foraminal according to anatomical landmarks such as medial edge of the articular facets and medial or lateral border of pedicles on axial plane.^7^ Diagnoses and classifications of disc herniation were performed by radiologists specialized in spine diseases. To investigate the clinical characteristics of foraminal and extra foraminal disc herniation compared with central and subarticular disc herniation, we established 2 groups: a medial group including central and subarticular disc herniation, and a lateral group including foraminal and extraforaminal disc herniation. Multiple disc herniations with foraminal disc herniation observed in MRI were included in the lateral group.

The 352 patients who were followed up for at least 12 months after completion of treatment were included in this study. Clinical characteristics such as age, gender, duration of pain, location of dominant pain (axial or leg pain), numeric rating scale (NRS) at pretreatment, number of disc herniations, severity of disc herniation (protrusion or extrusion), presence of leg muscles’ weakness, treatment method (conservative or surgical), and clinical outcome (successful or unsuccessful) were investigated. In terms of treatment method, patients who received only conservative management were regarded as conservative, whereas, those who underwent surgery due to failure of conservative management were defined as surgery. Successful clinical outcome was defined as 50% or more reduction of NRS after at least 12 months of treatment in comparison with pretreatment NRS.

These data were compared between the medial and lateral groups. In addition, clinical outcomes were compared in whole subjects as well as in the conservative and surgery subgroups.

### Statistical Analysis

The SPSS version 14.0 statistical package (SPSS Inc., Chicago, IL) was used for statistical analysis. Chi-square test was used to compare gender proportions, location of dominant pain (axial or leg pain), number of disc herniations, severity of disc herniation (protrusion or extrusion), presence of leg muscles’ weakness, treatment method (conservative or surgical), and clinical outcome (successful or unsuccessful) between the lateral and the medial groups. Student's *t*-test was performed to compare differences in age, duration of pain, and pretreatment NRS between the 2 groups. Results were considered statistically significant if the *P* value was less than 0.05.

## RESULTS

Among the 352 patients, the lateral group included 74 (21%) patients and the medial group included 278 (79%) patients. Mean age of the lateral group was significantly higher than the medial group. The lateral group showed significantly larger proportion of patients with radiating leg pain and multiple levels of disc herniations than the medial group. No significant differences were found in terms of gender proportions, duration of pain, pretreatment NRS, severity of disc herniation, and presence of leg muscles’ weakness. The proportion of patients who underwent surgery was not significantly different between both groups. However, the proportion of patients who accomplished successful pain reduction after treatment was significantly smaller in the lateral than the medial group (Table 1).

**TABLE 1:**
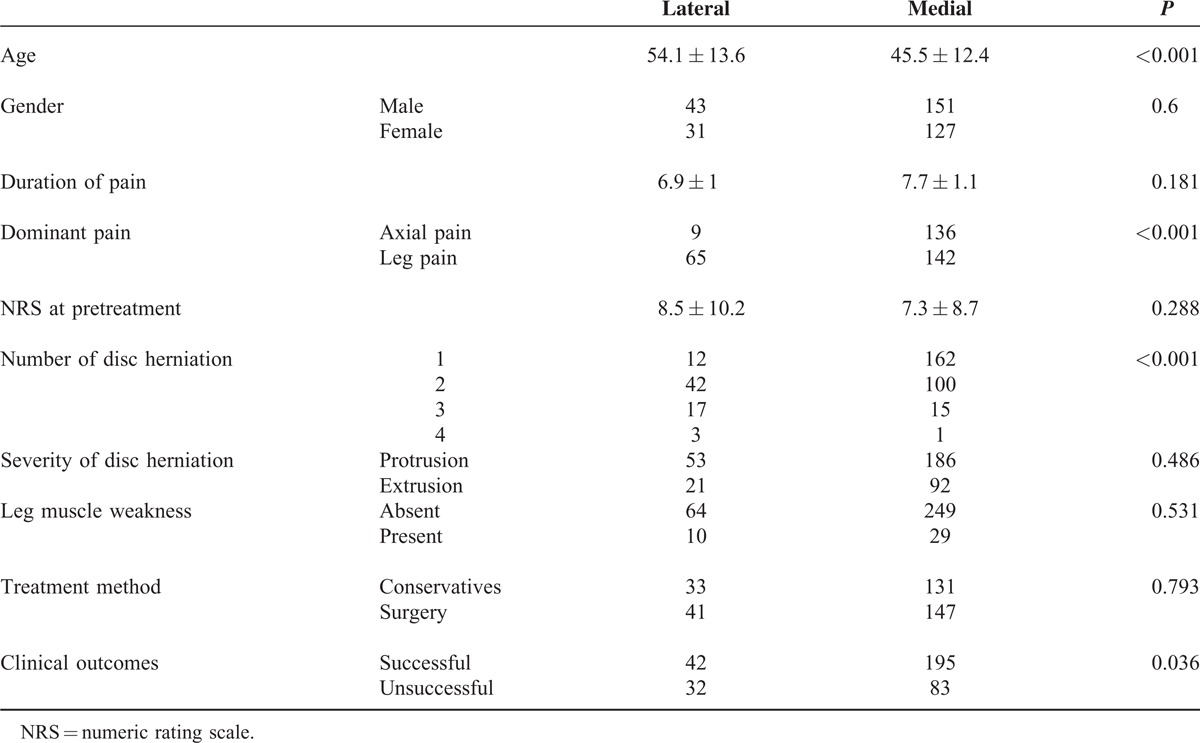
Comparison of Clinical and Radiological Characteristics Between Lateral and Medial Disc Herniation

In terms of conservative and surgery treatment subgroups, the lateral group showed trends toward poorer outcomes than the medial group, which was statistically insignificant (Table 2).

**TABLE 2:**
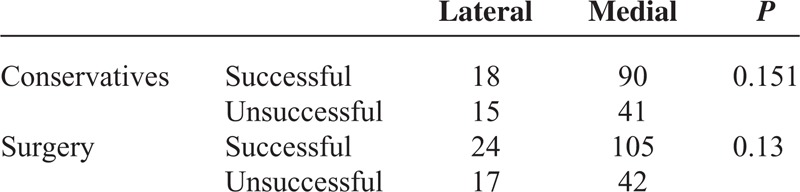
Comparison of Clinical Outcomes According to Treatment Method (Conservative Managements and Surgery) Between Lateral and Medial Disc Herniation

## DISCUSSION

In this study, the ratio of lateral to medial disc herniation was approximately 1:4. The proportion of lateral disc herniation in our study was relatively higher than in other reports, which mentioned that lateral disc herniation accounted for approximately 7% to 12% of lumbar disc herniations.^1,2,8^ This might be because the criteria of our study were different and stricter than those of other studies. We selected only the patients with localized disc herniation (less than 25% of circumference) as suggested in recent recommendations.^9^ In addition, we excluded migrated and sequestrated disc herniation, which could have resulted in more decrease in the number of medial disc herniation in comparison with lateral disc herniation. The way of classification of multiple disc herniations also contributed to larger proportion of lateral disc herniation because if at least 1 of the multiple disc herniations was determined as foraminal or extraforaminal, it was included in the lateral group.

Our study demonstrated that lateral disc herniation was more associated with multiple disc herniations. This result was unique to our study, as to our knowledge, the relationship between lateral disc herniation and multiple disc lesions has not been presented before in the literature. We postulated that disc degeneration and herniation could increase the instability of the adjacent disc segment, especially in rotational or lateral bending motion, which caused lateral side annular tearing. A biomechanical study identified that 1 level disc herniation increases the range of translational and angular motion at the adjacent level, but not to the degree of significant instability.^10^ However, this study included 1 level of disc herniation and observed only angular and translational motion in the sagittal plane, without including rotational or lateral motion. Other studies presented results that could support our assumption. Two level disc degenerations caused more segmental hypermobility of adjacent disc levels during lateral bending and twisting than during flexion-extension.^11^ Disc degeneration caused marked segmental instability, which was more prominent in lateral bending than flexion-extension.^12^ Two studies about spondylolisthesis and its associated disc herniation pattern stated that spondylolisthesis increases susceptibility of lateral disc herniation by increasing lateral instability. This suggests that segmental instability is more closely related to lateral instability and lateral disc herniation.^13,14^ One biomechanical study revealed that combined motions of lateral bending, flexion, and axial rotation caused tear from the nucleus to posterolateral annulus, which suggested that unstable and asymmetrical spinal motions could lead to lateral disc herniation.^15^ Our results could be explained by the combination of these mechanisms. Multisegmental disc problems produced motional instability of adjacent levels, especially in lateral and rotational motions, which could contribute to lateral annular rupture and consequently lateral rather than medial disc herniation.

Our study demonstrated that the mean age of lateral disc herniation was higher than that of medial disc herniation. This was also found elsewhere in the literature, but without an explanation.^4,5,16^ We assumed that older patients had more number of disc problems and lateral instability, which acted as a risk factor for producing lateral disc herniation.

Expectedly, our results showed that lateral disc herniation was more closely related to radiating leg pain. The article comparing lateral disc herniation with medial disc herniation indicated that lateral disc herniation involved more severe radicular leg pain and more frequent occurrence of sensory dysesthesia than medial herniations.^5^ Higher frequency of radicular leg pain in lateral disc herniation was because this type mechanically irritated or compressed the exiting nerve root or dorsal root ganglion inside of a narrow canal in more direct manner than medial disc herniation.^1,17,18^

It was against our expectation to find no significant difference in prevalence of clinical motor weakness between lateral and medial disc herniation groups, which was also stated elsewhere in other literatures.^1,5^ This might be because patients with lateral disc herniation visited the hospital due to radicular pain before neurologic deficits were clinically manifested or that lateral disc herniation lead to less injury of the ventral root, which produced less paresis of the muscle innervated by the motor neuron.^5^

Lateral disc herniation revealed poorer outcomes than medial disc herniation in the whole population of this study. In terms of individual treatment method – conservative and surgical – lateral disc herniation trended toward worse outcomes, although it was statistically insignificant. No difference existed between proportions of conservative and surgical treatment. This meant that the worse treatment outcome in lateral disc herniation was not a result of the specific treatment method. More severe or irreversible nerve damage, caused by direct compression within a narrow neural foramen, produced persistent pain even after treatment, irrespective of treatment method. Narrow space resulting from herniated fragment into root foramen prevented the medication from spreading effectively in spite of appropriate trans foraminal epidural injection.^6^ Even appropriate surgical decompression could not help to restore normal nerve function.^5,17,19^ In addition, poor outcome of lateral disc herniation could be explained by the fact that it was more associated with multiple levels of disc herniation or older age which were poor prognostic factors.^5,20^ However, 1 study comparing lateral and medial disc herniation without foraminal stenosis reported that the outcome of lateral herniation was not worse than that of medial disc herniation.^4^ They explained that poorer outcome in lateral disc herniation stated in previous literature might be caused by the inclusion of patients with associated foraminal stenosis. Our study showed worse treatment outcomes of lateral disc herniation despite excluding spinal stenosis in patients’ selection process. The main cause of difference between that study and others – including our study – was the different definition of lateral disc herniation. They included subarticular disc herniation in the group of lateral disc herniation, which was the main difference from other studies’ definition, most of which regarded lateral disc herniation as foraminal and/or extra foraminal.^2,5,17,20^

The following were the limitations of this study. First, the study design was retrospective and the patients who were not followed up for 12 months were not included. Second, there might be criticism about the way of defining the study groups in case of multiple disc herniations. If at least 1 multiple disc herniation was foraminal or extraforaminal on MRI, this was included in the lateral group which could have increased its proportion. This was because we intended to clarify the relationship of multilevel disc herniations with the occurrence of lateral disc herniation; therefore, we focused on the presence of lateral disc herniation irrespective of the coexistence of medial disc herniation. Thus, if at least 1 level of lateral disc herniation was observed among multiple disc herniations, we defined this as the lateral group and found that occurrence of lateral disc herniation was more associated with multiple disc herniations.

## CONCLUSION

Patients with lateral disc herniation were older and had larger proportion of radiating leg pain than those with medial disc herniation. Lateral disc herniation was associated more with multiple disc herniations and worse clinical outcomes after treatment than medial disc herniation.
